# Hospital-at-Home: The Good, the Bad, and the Ugly

**DOI:** 10.1089/pop.2023.0211

**Published:** 2023-11-24

**Authors:** Pouya Afshar

**Affiliations:** Presidium Health, San Diego, California, USA.

As of August 2023, the COVID-19 pandemic claimed the lives of nearly 7 million people worldwide, leaving a dark shadow in every corner of the globe.^1^ Beyond the grim aftermath, the pandemic also graced us with some silver linings. The social isolation gave society a deeper appreciation of togetherness and the importance of prioritizing time with our loved ones. As air travel and personal transportation came to a grinding halt so did our carbon footprint, dramatically improving air quality and revitalizing our ecosystem, even if for a brief moment in time. The job market immediately pivoted as many industries came to the realization that the workforce can efficiently function in a remote setting.

Scientific advancements occurred at warp speed as we witnessed the fastest vaccine rollout in history.^2^ In the health care industry, COVID-19 was the catalyst that fostered the digital health revolution, the rapid adoption of telemedicine, and cultivated mainstream acceptance of home-based care models, including the Hospital-at-Home (HAH) movement. The surge of COVID-19 patients and the strain it placed on hospital bed capacity pushed the Centers for Medicare and Medicaid Services (CMS) to take unprecedented steps to allow hospitals to render services outside their facilities, paving the way for HAH. The HAH model, as the name suggests, allows for patients to receive medical care and treatment in the comfort of their own homes as opposed to being admitted to a traditional hospital.

## The Good

In the United States, HAH has been in existence for over 20 years with Dr. Bruce Leff serving as one of the pioneers who championed HAH at Johns Hopkins University. Some of the early HAH studies showed a 38% reduction in hospital costs, lower 30-day readmission rates (7% vs. 23%), and higher patient satisfaction.^3^ Despite these favorable metrics, the prepandemic HAH model was restricted primarily due to limited reimbursement. As a response to the COVID-19 Public Health Emergency, CMS passed waivers and provided diagnosis-related group (DRG) reimbursement to hospitals engaged in the HAH model. This was a pivotal milestone as DRG payments were historically tied to facility-based hospital services.

By November 2020, Mount Sinai Health System in New York became the first hospital approved for the HAH waiver program. Shortly thereafter 5 other health systems, including Harvard-affiliated Brigham and Women's Hospital and Massachusetts General Hospital joined the list of CMS-approved HAH programs. Commercial interests soon entered the HAH marketplace and in May 2021, Mayo Clinic and Kaiser Permanente announced a $100 million strategic investment in Medically Home to expand their ability to offer HAH services.

The HAH floodgates ensued thereafter—as of July 2023, the HAH Users Group lists 108 health systems actively providing HAH services and another 358 entities in the planning stages of a HAH program.^4^ This rapid uptick in HAH participation can be explained by (1) access to a readily available on-demand inventory of hospital beds at home, (2) a favorable DRG-payment model, and (3) pandemic tailwinds that swayed society's acceptance of telehealth and the delivery of health care services at home.

The clinical model behind HAH is predicated on patients arriving in the emergency department (ED), receiving the standard litany of diagnostic tests, and giving patients who meet inpatient criteria the option of transferring home for continued care. Similar to a traditional hospital admission, HAH beneficiaries require daily rounding by a medical provider and a clinical team to monitor their needs on an ongoing basis. Telehealth, remote patient monitoring devices, portable diagnostic/imaging equipment, and other digital health applications have played an integral role in managing the logistics of inpatient care at home. The technology, boots-on-the-ground clinicians, and other vital components of executing HAH are typically outsourced to vendors that have developed turnkey solutions.

## The Bad and The Ugly

From the outset, HAH has been touted as the savior that came to the rescue at a time when the pandemic exposed the supply chain weaknesses in our health care system. HAH addressed the limitations in bed capacity, produced better clinical outcomes, lowered readmission rates, improved patient satisfaction, all while reducing the total cost of care.

Despite many of these perceived benefits, there are some glaring concerns behind the HAH model. The rate-limiting step for HAH is admission to an ED, preventing non-hospital entities from participating in HAH. The launch of the HAH campaign was facilitated by the financial backing of the American Hospital Association (AHA), 1 of the top 3 lobbying groups in the United States, doling out over $500 million in the past 25 years to promote its interests.^5^

The AHA introduced their HAH campaign at a time when the health care system was on its knees, successfully persuading CMS to allow hospitals to reap the financial benefits of an exclusive agreement. CMS granted hospitals the same facility-based DRG reimbursement for non-facility-based HAH services, ushering in more than $8000 on average per DRG encounter.^5^ When this lofty facility-based DRG reimbursement is applied to HAH patients, the overhead and expenses are markedly reduced, translating into higher profit margins.

By removing the ED as the safety net for HAH recipients, opportunities exist for patients to be triaged and managed upstream. In the traditional HAH model, patients who experience an acute event are transferred to the ED and triage services are rendered. For the overwhelming majority of patients deemed to be hemodynamically stable (ie, do not require ICU level of care) and meet the loosely defined criteria for inpatient admission, they become eligible to return home under the HAH program.

**FIG. 1.**
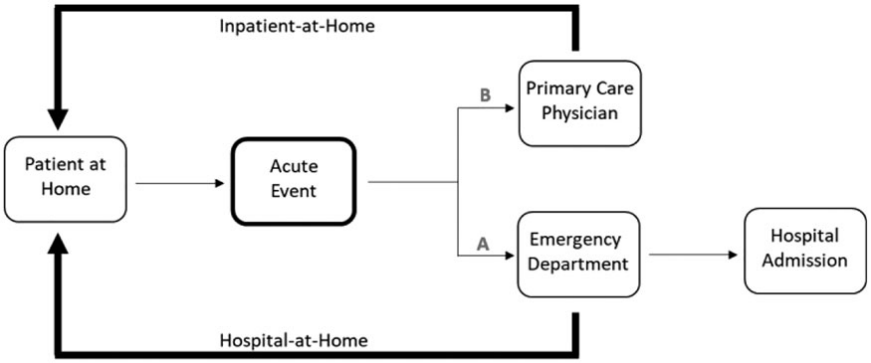
In the Hospital-at-Home model, patients who experience an acute event first receive care in the emergency department (“A” pathway). For patients who meet inpatient criteria, the traditional pathway would lead to a hospital admission. In the Hospital-at-Home model, patients receive their workup in the emergency department and are then diverted to receive inpatient services at home. Alternatively, an acute event can trigger the involvement of a primary care physician (“B” pathway). The primary care physician can bypass the emergency department, triage the patient, and render Inpatient-at-Home services in a similar manner to Hospital-at-Home, but without the preemptive emergency department encounter.

In an alternative “Inpatient-at-Home” (IAH) model, the same patient experiencing an acute event can be triaged by a non-facility-based medical team (ie, the primary care provider) while receiving the same HAH services (Fig. 1). With this alternative IAH model, patients never leave their home and the DRG payments are replaced with markedly reduced professional service fees ($8281 for a DRG payment vs. $717 for professional service fees).^6^

Pundits, including the majority of primary care physicians, would argue that the current outpatient primary care practice models do not provide the infrastructure nor the incentives to execute an IAH service. To successfully support an IAH program, primary care providers would have to radically supplement their practice model. Even with the telehealth applications adopted by many postpandemic clinicians, primary care providers would have to modify their practices (items 1–7, listed below) to effectively bypass an ED encounter and deliver services at home:

1. Provide 24/7 access
2. Triage patients with an acute event
3. Perform diagnostic services (ie, imaging and laboratory studies) at home
4. Render acute treatment at home, including delivery of medications
5. Provide clinicians to perform face-to-face services at home
6. Ability to perform steps 1–6 in an expedited manner
7. A shift from fee-for-service/capitated reimbursement to a performance-based payment model.

Perhaps the most critical component to the success of an IAH program is the willingness of the primary care providers to integrate acute care into their chronic-care-focused practices. For many years primary care physicians functioned as a longitudinal one-stop shop, managing patients over time, scheduling patients in a clinic, rounding on their patients in the hospital, providing house calls, and in most cases being accessible 24/7. Specialization occurred in the middle of the 20th century and began to limit the role of the primary care provider.

The current divide of primary care into chronic versus episodic acute care took shape in the mid-1990s with the introduction of hospitalists as a specialty of primary care. With these changes, chronic disease management became the domain of outpatient primary care practices, whereas episodic inpatient care shifted to the hospitalists. Although many will argue the hospitalist movement had a positive impact on the field of primary care, it also served the interests of the hospital and shifted more patients into the ED and ultimately into a hospital bed. It took a pandemic to spawn another subspecialty of primary care, cleverly disguised as HAH, aligning more of the hospitals' interests, creating an endless supply of heads-in-beds at home, and further diminishing the role of the primary care provider.

Not all hope is lost. As hospitals have continued to push their agenda, the Affordable Care Act has also created a ground swell of value-based care, pivoting away from fee-for-service to performance-based payment models. Although value-based care is still in its infancy, there has been renewed interest in expanding the role of primary care providers, incentivizing them with models that promote accessibility, the integration of acute and chronic care, and payments that are tied to clinical and financial performance. There are now countless examples of innovative value-based medical practices that have emerged, allowing primary care physicians to recapture their longitudinal care model in a manner that holds them accountable throughout a patient's health care journey.

These value-based practices blur the lines of acute and chronic care, and in many cases even incorporate elements of social care, making the jargon of HAH obsolete, as health care continues its shift away from institutions and into the home.^7^ For now, HAH remains under the exclusive control of hospitals backed with a DRG payment model. With the momentum of value-based care, it is not unreasonable to imagine a more level playing field for future HAH participants, including those not affiliated with a hospital system.

## Author Disclosure Statement

No competing financial interests exist.

## Funding Information

No external funding was received for this article.
